# Seasonal Preservation Success of the Marine Dinoflagellate Coral Symbiont, *Symbiodinium sp*.

**DOI:** 10.1371/journal.pone.0136358

**Published:** 2015-09-30

**Authors:** Mary Hagedorn, Virginia L. Carter

**Affiliations:** 1 Department of Reproductive Sciences, Smithsonian Conservation Biology Institute- National Zoological Park, Front Royal, VA, United States of America; 2 Hawai'i Institute of Marine Biology, University of Hawaii, Kaneohe, HI, United States of America; King Abdullah University of Science and Technology, SAUDI ARABIA

## Abstract

Coral reefs are some of the most diverse and productive ecosystems on the planet, but are threatened by global and local stressors, mandating the need for incorporating *ex situ* conservation practices. One approach that is highly protective is the development of genome resource banks that preserve the species and its genetic diversity. A critical component of the reef are the endosymbiotic algae, *Symbiodinium sp*., living within most coral that transfer energy-rich sugars to their hosts. Although *Symbiodinium* are maintained alive in culture collections around the world, the cryopreservation of these algae to prevent loss and genetic drift is not well-defined. This study examined the quantum yield physiology and freezing protocols that resulted in survival of *Symbiodinium* at 24 h post-thawing. Only the ultra-rapid procedure called vitrification resulted in success whereas conventional slow freezing protocols did not. We determined that success also depended on using a thin film of agar with embedded *Symbiodinium* on Cryotops, a process that yielded a post-thaw viability of >50% in extracted and vitrified *Symbiodinium* from *Fungia scutaria*, *Pocillopora damicornis* and *Porites compressa*. Additionally, there also was a seasonal influence on vitrification success as the best post-thaw survival of *F*. *scutaria* occurred in winter and spring compared to summer and fall (P < 0.05). These findings lay the foundation for developing a viable genome resource bank for the world’s *Symbiodinium* that, in turn, will not only protect this critical element of coral functionality but serve as a resource for understanding the complexities of symbiosis, support selective breeding experiments to develop more thermally resilient strains of coral, and provide a ‘gold-standard’ genomics collection, allowing for full genomic sequencing of unique *Symbiodinium* strains.

## Introduction

Coral reefs, some of the oldest and most diverse ecosystems on our planet, are under siege. Climate change is contributing to bleaching and acidification, that leads to stress and degradation, while the anthropogenic effects of increased sedimentation, nutrient over-load, excessive fishing and pollution have collectively caused a widespread, well-recognized reef crisis [1–6]. Approximately 54% of all coral reefs are threatened by local and global stressors [7] with ~70% of marine fish stocks fully- or over-exploited [8]. Noteworthy is that herbivorous fish, that help maintain the balance of the reef, are reduced 50% globally [9]. Although well-managed marine parks afford some protection, nevertheless reefs generally still remain hyper-vulnerable to disease, natural disasters and human impacts. Because these threats do not respect geo-political boundaries, we must implement multiple protection and preservation strategies, including *ex situ* conservation.

This paper is focused on one of the more strategically-important elements of the reef, the endosymbiotic dinoflagellates, *Symbiodinium sp*. that are believed to confer adaptive resiliency to coral. Throughout the world’s oceans, *Symbiodinium* are currently subdivided into 9 distinct clades (A to I) [10–12], but we concentrated on those in Hawaii. LeJeunesse et al. [13] used molecular tools to analyze the diversity of the *Symbiodinium* inhabiting Hawaiian coral and found 10 distinct symbiont types from eight coral species (nine in clade C subtypes and one in clade D). There was no clear dominant generalist symbiont in Hawaiian coral, as in the western Pacific or Caribbean, where a few generalists inhabit many coral species [13]. But that is not the entire story, studies [14–18] suggested that the coral/symbiont partnership may more flexible than previously believed, especially in areas at a latitudinal extreme, like Hawaii. Flexibility of symbiosis in coral clade preference is now observed at many levels, from changes in clades during development [19–22], to multiple clades inhabiting microhabitats in single colonies [23, 24], to post-bleaching adaptations and switching clades [10, 25], and conferring adaptive resiliency to the coral, such as greater thermal tolerance to warming oceans [10, 14, 25, 26]. Others corals do not switch or shuffle their symbionts, yet appear to be thermally tolerant [27, 28]. Because coral appear to host multiple clades simultaneously, this may allow them modest acclimatization to changing ocean conditions [15]. Symbionts living in more marginal areas may prove essential in understanding how coral reefs recover from environmental perturbations, and a wide diversity of symbionts should be included into the designs of coral Marine Protected Areas [10], and by extension, into genetic banks.

There are several marine algal culture collections around the world, including the Provasoli-Guillard National Center for Culture of Marine Phytoplankton (US) and Culture Collection of Algae and Protozoa (UK). These resources include multiple types of *Symbiodinium* managed in serial live-cultures, a practice that is expensive and labor-intensive due to the requirement of costly media and sterile conditions [29, 30]. Therefore, we explored an alternative approach to live-culture for preserving these unique biological materials through cryopreservation.

Although freezing is fairly well established for many marine algae [31, 32], this is not the case for the marine dinoflagellates, probably due to their oil-rich composition [33], which is also a challenge in cryopreserving similar terrestrial plant counterparts [34]. However some cryopreserved *Symbiodinium* cultures exist within the aforementioned culture collections, but the post-thaw expansion culture time can take months [32, 35], suggesting that the cryopreservation process for these cells is not well understood. Cryobiological theory holds that the successful freeze thawing of any cell first requires some intracellular water to be removed and at least partially replaced with a non-toxic cryoprotectant that prevents intracellular ice formation and cell lysis. As any cell is slow-cooled, stress is imposed by the chilling temperature and the cryoprotectant itself that has the potential of becoming toxic as water exits the cell and the solutes become more concentrated [36]. In previous work, we examined the cryosensitivity of *Symbiodinium* to both chilling temperatures and a wide range of solutes [37]. These previous findings revealed that standard slow-freezing cryopreservation may be unsuccessful for *Symbiodinium* due to their high sensitivity to chilling, osmotic stress and cryoprotectant toxicity.

Our pilot work for the present study supported this view. Specifically, *Symbiodinium* extracted from two Hawaiian species, *Fungia scutaria* and *Porites compressa*, were cryopreserved using standard slow-freezing rates of 0.1, 1, 3, 20 or 100°C/min, all of which resulted in no post-thaw viability (defined as having an intact photosystem after thawing as measured with a pulse amplitude fluorometer). One of the more important uses of cryopreserved *Symbiodinium* might be using different clades to help the coral survive changing conditions. Toward that goal, 4-day old *F*. *scutaria* coral larvae initially took up native extracted, cryopreserved and thawed native *Symbiodinium* type C1f, but 2 days later had eaten or rejected these cells, suggesting there was something wrong with them.

The combined observations that these *Symbiodinium* species appeared chill- and cryoprotectant sensitive as well as rejected by the coral host upon thawing clearly indicated the need for a more novel preservation approach. Vitrification, a non-equilibrium, ultra-rapid cooling technique, appeared prudent to examine as this procedure ‘outruns’ the chilling sensitivity element for most cells while often providing improved survival for many cell types as well as embryos [38]. The major factors involved in vitrification are: 1) the stepwise addition of highly concentrated solutions of cryoprotectants and sugars that dehydrate the target cell; and 2) ultra-rapid cooling of the cell suspension (e.g., 1,000°C/min) to form a transparent glass-state rather than ice crystals [38]. Over the past 30 years, traditional methods of vitrification have transformed assisted reproductive techniques for handling human embryos which are now vitrified with a post-thawing success of >90% [39]. Until recently, human eggs were not well preserved by traditional vitrification processes because of a high lipid content imposing extreme sensitivity to chilling temperatures [40]. Wedding classical vitrification protocols with the ultra-fast thawing rates (e.g., 10,000°C/min) of new films, called Cryotops has now made human oocyte cryopreservation highly successful [40]. Like human oocytes, *Symbiodinium* contain an abundance of lipids [41] that may be contributing to their chilling vulnerability [37]. Thus, vitrification may help with cryopreserving *Symbiodinium*, especially as new cryo-cocktails are now available that form excellent glassy states that, in turn, may reduce toxicity and osmotic stress.

Our goal was to elucidate the biological mechanisms required to successfully cryopreserve *Symbiodinium* extracted from three Hawaiian coral species, specifically *F*. *scutaria*, *P*. *compressa* and *Pocillopora damicornis*. *Symbiodinium* from *F*. *scutaria* was used as a model for triaging a suite of candidate cryoprotectants and vitrification protocols that were maintained post-thaw over 90 min. Based on those results, we developed a set of vitrification protocols that allowed *Symbiodinium* to survive for at least 24 h post-thaw. Then, the best candidate protocol was tested throughout the year to determine a potential role of seasonal variation on the success of the *Symbiodinium* to withstand freezing. Additionally, the candidate protocol was tested side-by-side with accepted slow-freezing methods [35] for *Symbiodinium* to compare their post-thaw results at 24 h. And finally, to determine the applicability of our process across species, we conducted a comparative vitrification study exposing the *Symbiodinium* from *F*. *scutaria*, *P*. *compressa* and *P*. *damicornis* to our candidate vitrification protocol, assessing their 24 h post-thaw viability.

## Materials and Methods

### Coral Collection and Husbandry

Whole individuals of *F*. *scutaria*, colonies of *P*. *damicornis* and fragments of *P*. *compressa* were collected from the shallow reef flats around Coconut Island in Kaneohe Bay, Hawaii from 2011 to 2015. Different locations on the reef flat were chosen for collection to ensure as much coral genetic diversity as possible. Coral were collected from the Bay and used immediately or maintained in flowing seawater tables connected directly to Kaneohe Bay with natural temperature and light exposure throughout the study. Collection was performed with the appropriate permits from the state of Hawaii’s Department of Land and Natural Resources (Special Activity Permit # SAP 2011: 2011–1, 2012: 2012–63, 2013: 2013–47 and 2014: 2015–17). No institutional ethical approval was required for any of the experimental research described herein.

### Monitoring and Preparing *Symbiodinium* Samples

*Symbiodinium* were extracted from live coral tissue, cleaned and their health monitored according to the methods of Hagedorn et al 2010 [37]. Briefly, a Junior Pulse Amplitude Modulated fluorometer (Junior-PAM, Walz, Germany) provided an indication of the viability of photosynthesis in Photosystems I and II. Settings on the Junior-PAM, specifically the gain and intensity settings, were kept constant throughout each and across all experiments in these studies to ensure that we were observing physiological changes. The percent change from a sample’s initial quantum yield post-treatment was reported. For the purpose of this paper, any quantum yield or Y-value at or below 0.050 was defined as non-viable and as having a non-functional photosystem [37]. By inference, quantum yields with the least change from their initial measures, indicate a robust and healthy *Symbiodinium* sample, whereas those close to 100% change were considered dead. Measurements with the Junior-PAM were conducted under consistent fluorescent lighting in the laboratory at mean photosynthetically active radiation (PAR) value of 4.6 ± 0.51 SEM μmols/m^2^/s (n = 5, Apogee Instruments, Model MQ-200). All solutions, including those containing the extracted *Symbiodinium*, were made in and maintained in 0.2 μm- filtered seawater (FSW), unless otherwise stated.

**Fig 1:**
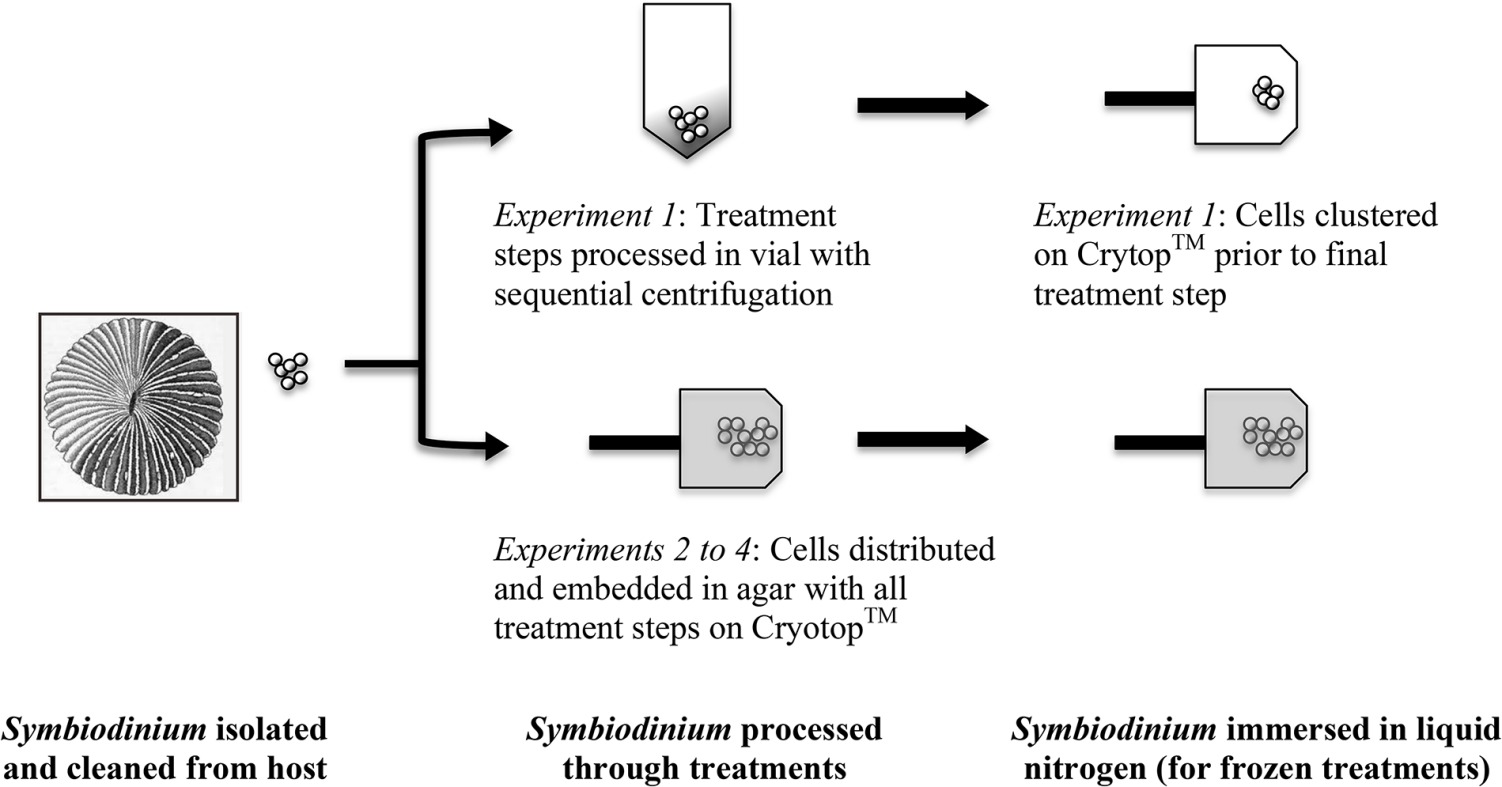
Schematic of cryopreservation methods used in all these experiments in this study.

Cryotop (Kitazato Corporation, Tokyo, Japan) methods have been used to manipulate human oocytes for ultra-rapid warming to produce successful vitrification [40]. To determine the applicability of these devices to cryopreserving *Symbiodinium*, we used a 2-μl drop of zooxanthellae on the Cryotop (Experiment 1 only) or a 1.5 μl drop spread as a thin agar film the following four treatments with *Symbiodinium* embedded within as mentioned in Fig 1 (used in all other Experiments) melted on to the top of the Cryotop. Conditions (i.e. temperatures and times for embedding) are given below in part 2. With this agar-and-*Symbiodinium* film method, the *Symbiodinium* did not fall off during the passage from solution-to-solution and during freezing or culture because they were embedded within the agar, making *Symbiodinium* manipulations easier. The conditions (i.e. temperatures and times for embedding) are given below in part 2. The agar-and-*Symbiodinium* film covered an area of the Cryotop approximately 0.5 cm in length from the tip of the Cryotop surface and had a domed shape (see schematic in Fig 1). During these protocols, *Symbiodinium* health was assessed after loading into agar and onto a Cryotops and then again during certain intervals post-thaw. Cryotops samples were moved through solutions and afterwards set into shallow dishes to await viability assessment with the PAM.

### Experiments

#### Experiment 1: Vitrification of *Symbiodinium* in a spherical pellet

Our previous studies [37] demonstrated that a 10% dimethyl sulfoxide solution (a penetrating cryoprotectant) appeared to minimize toxicity for *Symbiodinium*, while requiring about 60 min to equilibrate within the cell. To increase osmolality of the cryoprotectant, 0.5 M glucose or trehalose (as a non-penetrating cryoprotectant that helped dehydrate the cell by removing intracellular water) was added to 10% V/V dimethyl sulfoxide (to help stabilize and protect membranes). Therefore, all subsequent trials in this experiment used dimethyl sulfoxide (to help stabilize and protect membranes) often in tandem with 0.5 M glucose or trehalose.

*Symbiodinium* were extracted and cleaned [37], counted with a hemocytometer, and suspended in the following four treatments at a concentration equaling ~5 x 10^7^ cells/ml. The *Symbiodinium* were partitioned into four treatment subsamples and their initial quantum yield measured. These treatments were (1) FSW, (2) FSW + freezing, (3) CPA = cryoprotectant exposure, no freezing, and (4) Vitrification = cryoprotectant exposure + freezing. Six different vitrification protocols (defined below) were tested as the treatment 4. In all experiments the solutions and cryoprotectants used for treatment 3 and 4 were the same, except treatment 3 was not vitrified. All of the vitrification protocols had the same first step, which was a 10% dimethyl sulfoxide solution for 1 h followed by centrifugation (Eppendorf Centrifuge 5415D at 5900 x g for 5 min). Then the cells were resuspended in dehydrating step 2 solutions which differed and were either: 1) 10% dimethyl sulfoxide (5 min exposure); 2) 10% dimethyl sulfoxide + 0.5 M trehalose (5 min exposure), 3) 10% dimethyl sulfoxide + 1 M trehalose (5 min exposure), or 4 to 6) 10% dimethyl sulfoxide + 0.5 M trehalose + 0.5 M glucose (for 5, 10 or 15 min exposures). After step 2, a 2 μl drop of *Symbiodinium* and solute containing 1 x10^5^ cells was placed onto a Cryotop and immediately immersed in liquid nitrogen until the boiling stopped (Fig 1, upper schematic). Once frozen, the cells and Cryotops were immediately moved to 98 μl of FSW (23 to 25°C) in a 1.5 ml plastic tube. During freezing and warming, the *Symbiodinium* drop situated on the Cryotop was observed visually a few cm below the surface of all liquids for the presence of ice crystals (ice crystals form obvious white patterns viewable with the naked eye). Cells were maintained for 24 h in FSW. All samples were loosely covered over night to reduce stress from overt light input. However, the cover was typically removed about 15 min before the final assessment with the Junior-PAM. These samples were not dark-adapted. Once a sample’s Y-value was measured after a treatment, a percent change from its initial value was calculated.

#### Experiment 2: Vitrification of *Symbiodinium* in a laminar agar film

The design of Experiment 1 had three challenges. First, the *Symbiodinium* had to be centrifuged to transfer the cells from solution to solution potentially increasing cellular stress. Second, when the cells were thawed, the vitrification solution was only diluted 1:50, potentially adding to osmotic stress during the 24 h maintenance period. Third, the *Symbiodinium* were in a spherical profile, which increased the surface to volume ratio, potentially reducing permeation. To address these issues, we changed the *Symbiodinium* holding conditions to a laminar profile, thus reducing the surface-to-volume ratio. Specifically, we mixed *Symbiodinium* 1:1 in agar at different agar concentrations (1.0, 1.25, and 1.5%) and at various starting mixing temperatures (45, 55, and 65°C). We tested 24 h survival to ensure that the *Symbiodinium’s* photosystem remained intact and that they stayed on the Cryotop during all vitrification manipulations at a final concentration in the agar of 1 x 10^7^ cells/ml (n = 32, *F*. *scutaria*). The sample’s quantum yield was measured initially and then again after an agar-*Symbiodinium* Cryotop was exposed to its treatment solution and maintained for 24 h in FSW. A percent change from its initial value was calculated. As above, the samples were loosely covered over-night and assessed 24 h later.

To understand how the change in the quantum yield related to the percentage of *Symbiodinium* left alive in the sample, a live: dead curve was created, as in a previous study [37] that demonstrated a linear relationship between the percent live cells in the sample and normalized quantum yield. However the earlier samples were measured in suspension and not in a laminar film, and we did not know if this might have an affect on the PAM readings. Therefore, a parallel control experiment was conducted to understand how the quantum yield values related to viability of the *Symbiodinium* within the physical measurement parameters on the Cryotop (i.e., Cryotop agar films in 1 ml Eppendorf tubes with the Junior-PAM at a PAR of ~5 μmols/m^2^/s, 23 to 25°C). *Symbiodinium* from *F*. *scutaria* (n = 3) were extracted and cleaned [37]. *Symbiodinium* samples consisting of live: dead cells were created in a range of ratios from 1:0 (all alive) to 0:1 (all dead) and ratios in between (e.g., 10:1, 1:5, 2:3 and 4:1). Cells were killed by three cycles of microwave exposure followed by flash freezing and a final PAM assessment to ensure they were dead, and then the live and dead cells were mixed in known proportions. A starting sample (10^7^ cells/ml) was split into two sub-samples. The first sub-sample was embedded in agar while the second was left in suspension. A sample’s quantum yield was measured immediately and at 24 h. When the live:dead ratio was regressed against a normalized quantum yield, these two sub-samples produced the same linear results, as obseved previously [37] (P> 0.05, F = 0.0011, Linear Regression, see S1 Fig). Therefore, the physical parameters of the sample does not change the physiology of the *Symbiodinium* and generally a 50% change in the quantum yield values in these studies related to an approximately 50% reduction in viable cells in the sample.

Based on the results of Experiment 1, *Symbiodinium* extracted from *F*. *scutaria* (n = 4) were embedded in 1.5% agar on the Cryotop (as diagramed in Fig 1). As above, four treatments were assessed: (1) FSW, (2) FSW + freezing, (3) CPA = cryoprotectant exposure, no freezing, and (4) Vitrification = cryoprotectant exposure + freezing. Four different vitrification protocols were tested in treatment 4, as defined in Table 1. A candidate vitrification protocol was selected from these results bases on the percent change of the *Symbiodinium’s* quantum yield and used in subsequent experiments. The rationale for using trehalose in these experiments it that this sugar is produced and exported by *Symbiodinium* [42], so it may naturally be playing some sort of protective role. Methanol was chosen because it is non-toxic to *Symbiodinium* [37].

**Table 1:** Candidate vitrification treatments for *Symbiodinium*.

*ProtocolName*	^*#*^ *Step 3* * *Process*	*Thawing*
**Vitri-1**	10% DMSO+0.5M Trehalose +**5% Methanol**, 5 min	FSW
**Vitri-2**	10% DMSO+0.5M Trehalose +**7.5% Methanol**, 5 min	FSW
**Vitri-3**	10% DMSO+0.5M Trehalose +**5% Methanol**, 5 min	0.5 M trehalose, 20 min
**Vitr-4**	10% DMSO+0.5M Trehalose +**7.5% Methanol**, 5 min	0.5 M trehalose, 20 min

^#^ Step 3 was preceded by Step 1, a 10% dimethyl sulfoxide (DMSO) solution exposure for 1 h, and Step 2, a 10% DMSO + 0.5M Trehalose exposure for 30 min.

* After this Step 3 the Cryotop^T1M^ was immersed in liquid nitrogen until boiling ceased.

In Culture Collections the post-thaw expansion of *Symbiodinium* in culture can take months, which may be due to the low number of cells surviving slow freezing cryopreservation. In this experiment, we compared the slow-freezing method [35] employed by many culture collections and our optimal vitrification process (above) to understand whether vitrification produced substantial numbers of viable cells post-thaw. Thus, *Symbiodinium* from *F*. *scutaria* (n = 2) were extracted and divided into two sub-samples and exposed to (1) our candidate vitrification protocol (Vitri-3, described above), and (2) the slow freezing method [35]. As before, the vitrification and slow freezing sub-samples were divided again into our standard four treatments: (1) FSW, (2) Frozen, no cryoprotectant exposure, (3) CPA = cryoprotectant exposure, no freezing, and (4) CPA plus slow freezing or vitrification + freezing. For the slow-freezing process, four 1 ml samples of *Symbiodinium* (1 x 10^7^ cells/ml) were assessed initially with the Junior-PAM, centrifuged (Eppendorf Centrifuge 5415D at 900 x g, 5 min) to form a pellet, and the supernatant removed. Then, 1 ml of FSW was added to create treatments 1 and 2 (above) and 1 ml of 20% methanol was added to create treatments 3 and 4 (above). For treatments 2 and 4 designated for slow-freezing, the methanol was allowed to equilibrate into the cells for 10 min at 4°C. These samples were then placed into a -80°C freezer for 2 h, followed by immersion in liquid nitrogen for 10 min, bringing the samples down to -196°C. Each frozen sample was thawed by gentle agitation in room temperature water. At this stage, the cells were washed three times by concentrating them via centrifugation (900 x g, 5 min), removing the supernatant and supplementing them with1 ml of FSW. After the third wash, samples were covered with aluminum foil to block stray light and rested over night in FSW for at least 20 h. Then quantum yield values for each treatment were determined for the slow frozen and vitrified samples at the same time.

#### Experiment 3: Influence of season and the ability of *Symbiodinium* to survive vitrification

Findings from Experiment 2 identified the best candidate vitrification protocol for more detailed testing. As this experiment was being conducted during different times of the year, we had noticed variation in our quantum yields post-vitrification over time. We postulated that this variability might be due to seasonal changes in the physiological conditions of the *Symbiodinium*. Previous work on chilling sensitivity in coral fragments with intact symbionts [43] suggested that coral fragments were more tolerant to chilling in winter compared to spring [37], although this study was neither longitudinal nor conclusive. To determine the influence of season on the physiological response of *Symbiodinium* to vitrification, our candidate vitrification protocol (Vitri-3) was tested at least bi-monthly from February 2014 through January 2015. Specifically, we evaluated the change in the quantum yield after 24 h post-thaw from a minimum of five *F*. *scutaria* sampled per month throughout the year.

#### Experiment 4

As Experiments 1 to 3 were conducted exclusively by extracting *Symbiodinium* isolated from *F*. *scutaria*, we decided to test the applicability of the vitrification to other species, specifically to other *Symbiodinium* (of Hawaiian coral origin) isolated from *P*. *damicornis* (n = 3) and *P*. *compressa* (n = 3) in parallel with our model *F*. *scutaria* (n = 3). Samples collected during the winter, the *Symbiodinium* samples were embedded in agar, incorporated onto the Cryotop and immersed in liquid nitrogen. As above, four treatments were assessed: (1) FSW, (2) FSW + freezing, (3) CPA = cryoprotectant exposure, no freezing, and (4) Vitrification = cryoprotectant exposure (using protocol Vitri-3 in Table 1) + freezing. The 24 h maintenance in FSW post-thaw and viability analysis were the same as described in Experiment 2 and 3 above.

### Statistics

All data analyses were performed using Prism 6.0 (Graphpad, San Diego, CA) and Excel (Microsoft, Redmond, WA). All percentage change data were log transformed prior to statistical analyses. For multiple group comparisons, normality was tested graphically and ANOVAs with a Tukey’s, Bartletts or Dunnett’s Multiple Comparison post-tests, a Kruskal-Wallis, or Linear Regression were performed (all post-tests were specified in the Results), and all data are expressed in mean ± SEM.

## Results

### Experiment 1: Vitrification of *Symbiodinium* in a spherical pellet

None of the *Symbiodinium* exposed to the vitrification treatments described for this experiment survived; as there was no post-thaw quantum yield values > 0.05 after 24 h (Table 2). If the total osmolality of the solution was ≤ 3 M, the droplet failed to vitrify, potentially causing lethal intracellular ice damage. An increase in solution osmolality to > 3 M did not improve post-thaw quantum yield following *Symbiodinium* vitrification. Even extending the dehydration time (during Solute Step 2) failed to increase post-thaw viability (Table 2). Therefore, although the sample drop appeared grossly vitrified, there was no post-thaw viability, suggesting intracellular damage. The data also appeared to suggest that most treatments were imposing little toxicity in the *Symbiodinium*, because all solution exposures (CPA column, Table 2) had only modest changes in quantum yield values (range, 0 to 30%). As there also was no clear preference for either glucose or trehalose (Table 2), trehalose was chosen since it was naturally produced by *Symbiodinium* [42], and would be an appropriate test element for subsequent experiments.

**Table 2:** *Symbiodinium* change in quantum yield (%) after exposure to vitrification treatments and 24 h maintenance in FSW.

^*#*^ *Step 2*	*FSW*	*Frozen* ^*1*^	*CPA* ^*2*^	***Vitrified***	*Drop Vitrified?*
10% DMSO	11.0	85.5	32.9	88.9	No
10% DMSO+0.5 M trehalose(5 min)* 3,000 mOsm	6.4	85.5	25.8	96.0	No
10% DMSO+1 M trehalose(5 min) 3,500 mOsm	1.5	96.5	15.8	94.9	Yes
10% DMSO+0.5 M trehalose+0.5 M glucose (5 min) 3,500 mOsm	10.2	97.3	11.5	92.2	Yes
10% DMSO+0.5 M trehalose+0.5 M glucose (10 min) 3,500 mOsm	9.9	94.0	9.4	91.1	Yes
10% DMSO+0.5 M trehalose+0.5 M glucose (15 min) 3,500 mOsm	0	89.6	0	97.1	Yes

^#^ Step 2 was preceded by Step 1, a 10% dimethyl sulfoxide (DMSO) solution exposure for 1 h; Frozen^1^ = vitrified no cryoprotectant exposure; CPA^2^ = exposed to cryoprotectants, no freezing

* = exposure time to solute

### Experiment 2: Vitrification of *Symbiodinium* in a laminar agar film

**Fig 2:**
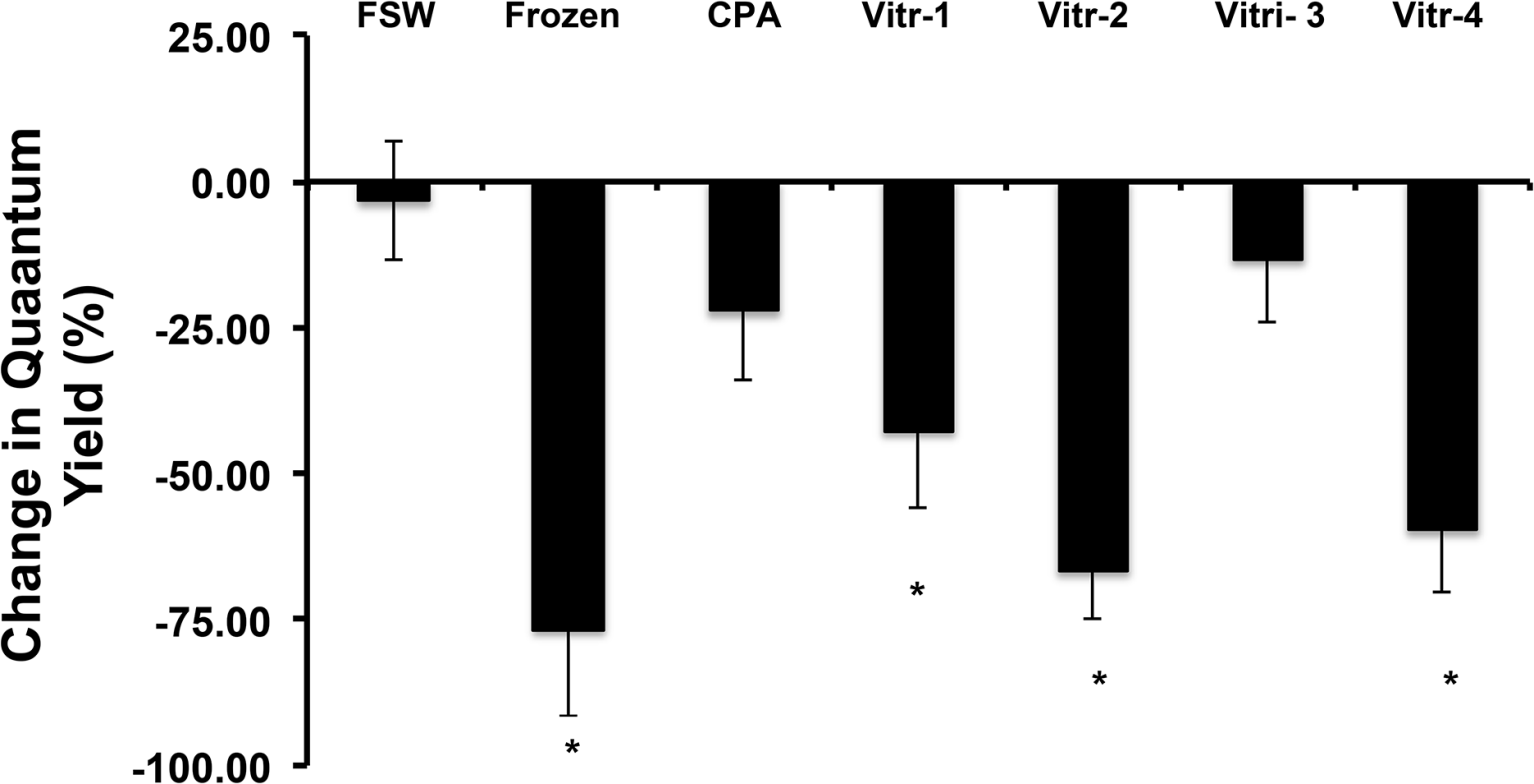
Influence of differing vitrification solutions on viability of *Symbiodinium*. Only the Vitri-3 solution produced viability results similar to the FSW control. Bars with an asterisk (*) are different from the control (P < 0.05).

When 1.0, 1.25, or 1.5% agar solutions were tested for the embedding process, we determined that only the 1.5% agar solutions remained consistently on the Cryotop throughout the stepwise vitrification procedure. During embedding, the agar had to be heated to liquefy. When we evaluated the agar mixing temperatures, there was no difference in overall percent change in the quantum yield after 24 h of any of the treatments (P>0.05; ANOVA, n = 25 to 32 *F*. *scutaria*, F = 0.147, Bartlett's test). All agar temperatures produced ~50% change exposures after vitrification (S2 Fig). Agar heated to 65°C adhered best and had the fewest failed treatments (6.3, 9.4, and 21.9% failed treatments for 65, 55, and 45°C initial agar heating, respectively). Therefore, the 1.5% agar solution heated to 65°C was chosen for all subsequent experiments. When Vitri-1 to Vitri-4 solutions were compared, only the Vitri-3 protocol produced viability results similar to FSW alone or CPA and vitrification solution but no freezing (Fig 2; P< 0.05, ANOVA, F = 21.63, Dunnett’s Multiple Comparison Test). Therefore, the Vitri-3 protocol was used in all further experiments. Moreover, using the same *Symbiodinium* samples only vitrification with Vitri-3 produced viable cells 24 h post-thawing, whereas slow freezing method [35] did not (S3 Fig).

### Experiment 3: Influence of season and the ability of *Symbiodinium* to survive vitrification

**Fig 3:**
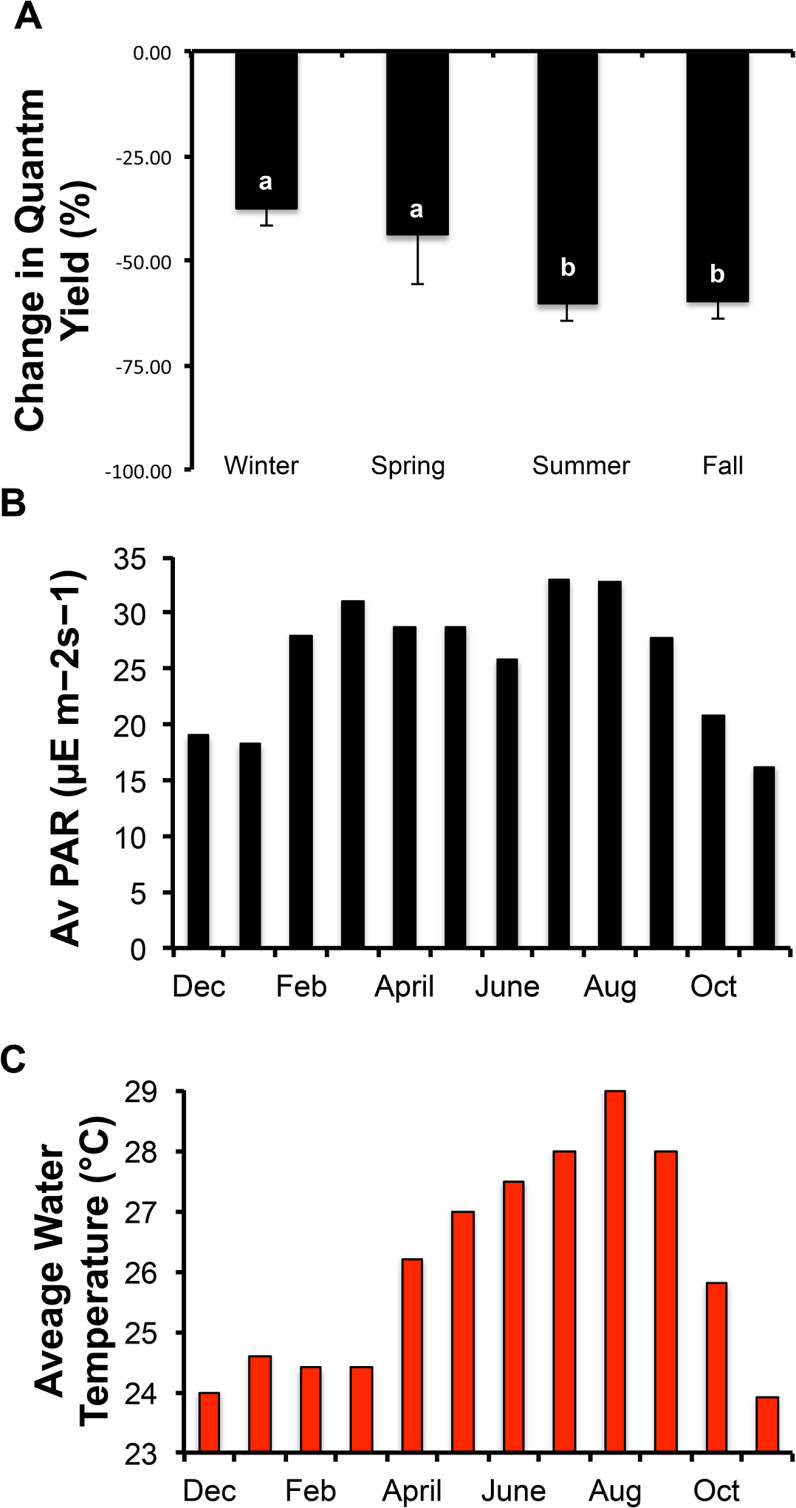
*Symbiodinium* and seasonal vitrification success. **A)** Winter and spring were optimal times for vitrification. Bars with differing superscripts were different (P < 0.05); **B)** A seasonal measure of PAR on Coconut Island, where winter months had the lowest light values (data from [44]); **C)** Seasonal temperature values on Coconut Island in 2014 where parts of fall and winter months had the lowest ambient temperatures.

When *Symbiodinium* samples were collected throughout the year and then vitrified with the single best protocol (Vitri-3), a seasonal pattern was revealed (S4 Fig). Samples collected in the winter month of February exhibited the most robust response to vitrification with a mean quantum yield change of 6.3% in 2014 and 22.5% in 2015. In a comparison example, samples from the summer month of June produced a mean quantum yield change of 70%. To understand the influence of time of year more clearly, months were clustered into seasons and compared with mean water temperature and PAR values on Coconut Island [44] (Fig 3). On average, there was a 37 and 44% change in quantum yield in the winter and spring, respectively, compared to 60% for both the summer and fall (P> 0.05, ANOVA, F- = 5.16; Tukey’s Multiple Comparison Test, Fig 3A). The environmental conditions that might influence seasonality in the *Symbiodinium* are PAR light level and water temperature at Coconut Island (Fig 3B and 3C).

### Experiment 4

**Fig 4:**
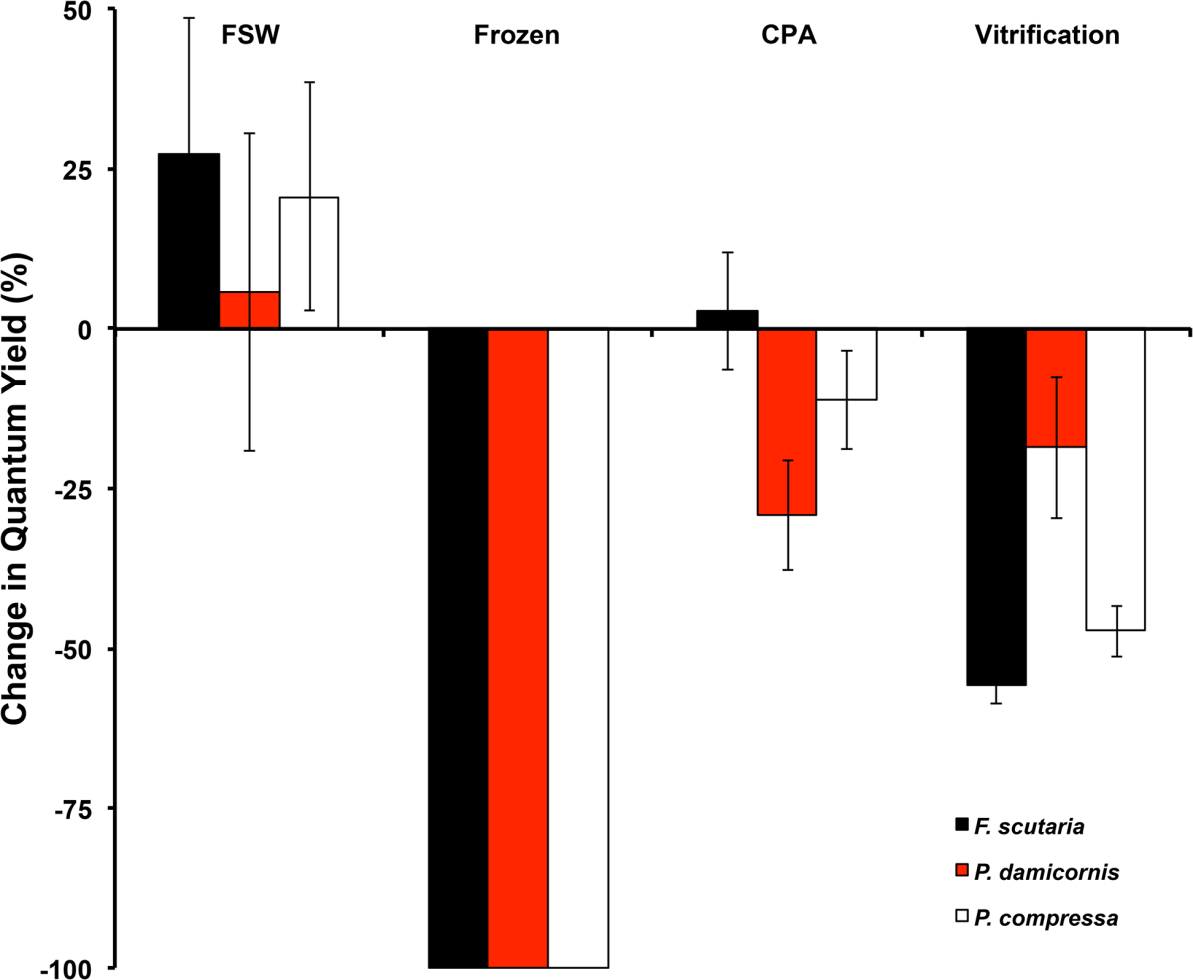
Comparative vitrification results, using the Vitri-3 treatment, for *Symbiodinium* from three Hawaiian coral species. At least ~50% survival was achieved regardless of source of the symbionts.

The Vitri-3 protocol using the Cryotops produced survival in the *Symbiodinium* of all three species tested. Survival in *P*. *damicornis* was higher (P < 0.05, Kruskal-Wallis test, Fig 4) with a mean change in quantum yield of 18.6%, compared to 56% and 47% for *F*. *scutaria* and *P*. *compressa*, respectively (P > 0.05, Kruskal-Wallis).

## Discussion

This study was designed to explore the efficacy of vitrification for the cryopreservation of the marine dinoflagellate coral symbiont, *Symbiodinium sp*.. These algal symbionts are essential for maintaining the health and viability of most warm water coral around the globe. During this study, we made several significant discoveries including: (1) the creation of a method to safely and quickly cryopreserve *Symbiodinium* by ultra-rapid freezing or vitrification; (2) the means to quickly assess the viability and estimate the number of viable algal cells 24 h post-thaw, using PAM fluorometry; (3) the finding that the success of this preservation method had a seasonal physiological component; and, (4) the successful efficacy of vitrification was applicable to *Symbiodinium* from multiple coral species. Thus, this protocol appeared capable of safely preserving the *Symbiodinium* of multiple coral species and, when collected during winter, resulted in a post-thaw survival of at least 50 to 90%, in the summer and winter, respectively.

Although a slow-freezing cryopreservation method exists for *Symbiodinium* [35], we postulate that few cells survive the slow-freezing process and may be the reason it takes months to culture them post-thaw by this method. In our comparison study (S3 Fig), vitrified samples produced a 58% change in quantum yield compared to 99% for slow freezing, indicating that the latter cell population was largely dead. If we compared the resulting numbers, and assumed that (1) ~40% of the cells were alive in the vitrified samples at 24 h and (2) the volume of the agar on the Cryotop was ~1.5 μl with an initial concentration of 10^7^ cells/ml, then we estimated that ~6.0 x 10^3^ were alive in the vitrified samples post-thawing and at 24 h. This is a large number to begin the culture process. By contrast, the number of cells alive in the slow frozen samples was below detection via PAM, so probably few cells remained alive.

The seasonal variation in the success of the vitrification process for *Symbiodinium* suggested that sampling and vitrification of *Symbiodinium* should occur ideally in winter. Other types of seasonal variation in physiological responses has been observed in lipid-rich microalgae grown outside [45]. Specifically, these microalgae show distinct shifts in their total lipids, found in the membrane and in vacuole, which were 11% in winter and 30% in fall, explained mostly by light and temperature [45]. We observed this same seasonal pattern of light and temperature correlating with the vitrification success in *Symbiodinium*. This seasonality is not surprising given that the amount of sunlight stimulating the symbiont’s photosystem is less in winter, potentially altering membranes or fat synthesis. In some animals, both the physiology and preservability of their germplasm can be seasonal [46, 47], as well. Additionally, cold hardiness produces significant changes to the extra- and intracellular physiology of somatic cells of plants [48] and animals [49, 50], allowing them to endure extreme cold during the winter months. For example, the mechanisms underlying cold-hardiness are now beginning to be understood for the frog [50], because as winter approached two cryoprotective agents, urea and glucose, are synthesized and mobilized to the cells to protect them from the chilling temperatures. Taken together, is seems reasonable that the *Symbiodinium* may produce more intracellular lipids during the high-light intensity and warm summer and fall months, thus increasing their chilling sensitivity and reducing their overall vitrification success. However, these physiological changes in lipid content remain to be tested to verify this mechanism.

Cryobanks reflect a new and major type of preservation that can be added to conventional museum archives, but in this case, the living biomaterials go beyond dried materials to include gametes, embryos, somatic cells, blood, and DNA. Most species’ genomic material, especially that of marine species, needs to be captured in the field far from sophisticated equipment. Vitrification is a field-friendly process that does not require a lot of equipment. Because these cells cannot often be captured and brought into the laboratory alive, cell preservation methods, such as cryopreservation, are paramount. During more standard preservation processes, such as immersion in alcohol, as is common for cells or tissues currently stored as museum specimens, DNA can often break into pieces over time [51]. This makes an extraordinarily strong and urgent case for creating cryobanks for cells, where they remain, frozen, but alive with near perfect genomic constructs. However, the future proof of concept for a global vitirified *Symbioninium* bank will be whether these cells expand in culture and are absorbed and maintained by coral larvae post-thaw. Both studies are currently ongoing.

Decisive conservation actions are needed to save reefs, with the first priority being habitat preservation. However, corals now face global rather than only local threats, requiring future-thinking tools, including *ex situ* conservation practices that can protect extant species and genetic diversity and integrity. In this context, we (and others) have argued for the biological banking of coral and their symbionts [52, 53]. This is critical in light of the ever increasing numbers of bleaching events since 1980 [3]. To date, only coral sperm can be cryopreserved [53] because of the large amounts of lipids in coral eggs and embryos, leading to chilling sensitivity inhibiting viability, but the ultra-rapid freezing of Cryotops, using cells in a more laminar distribution on the surface, hold great promise for out-running this sensitivity potentially allowing success in the future. A cryobank not only would serve to protect viable specimens of each high priority species, but also could be used to provide biomaterials to scientists for both basic and applied research. Meanwhile a portion of the bank could retain frozen but alive specimens for hundreds of years, if necessary, in liquid nitrogen, allowing the time necessary to mitigate threats and restore habitats. We also see thawed samples from the repository being used to ‘seed’ new gene diversity into damaged bleached reefs. Finally, it is noteworthy that oil-rich marine photosynthetic algae are the focus of multiple bio-prospecting ventures for new biofuels as alternate energy production [33] and would benefit from banking to reduce genetic drift of important strains.

As we face ever-warming summers around the world, bleaching has the potential to reduce coral numbers and diversity. Only through selective breeding or assisted evolution [54] will many species be able to tolerate the changing oceans, and flexible and thermally tolerant *Symbiodinium* may be key to this success. Forming a robust and comprehensive cryobank of the world’s *Symbiodinium* is critical for ensuring the maintenance of global coral diversity, will form a important tool for selective breeding experiments to develop more thermally resilient strains of coral, will create a greater understanding of symbiosis and will provide a ‘gold-standard’ genomics collection allowing full reads for DNA and RNA sequencing for these important cells.

## Supporting Information

S1 Fig Control PAM analysis of *Symbiodinium* cells demonstrating that the normalized quantum yields in specific live: dead ratios for cells embedded in agar and those in suspension produce similar results. (TIF)

S2 Fig Agar temperature effects on vitrification success of *Symbiodinium*. None of the temperatures tested affected quantum yield of the *Symbiodinium* after vitrification and 24 h maintenance in FSW (P > 0.05).
(TIF)

S3 Fig A comparison of the same *Symbiodinium* samples using vitrification (black bars) and slow freezing (grey bars). Only vitrification produces live cells post-thaw.
(TIF)

S4 Fig Seasonal variation in *Symbiodinium* response to vitrification. Winter months in Hawaii produced the best vitrification success.
(TIF)
