# Supplementary figures and images for: Seasonal Preservation Success of the Marine Dinoflagellate Coral Symbiont, Symbiodinium sp
Source: PLoS One. 2015 Sep 30;10(9):e0136358. doi: 10.1371/journal.pone.0136358 (PMC4589415; doi:10.1371/journal.pone.0136358)

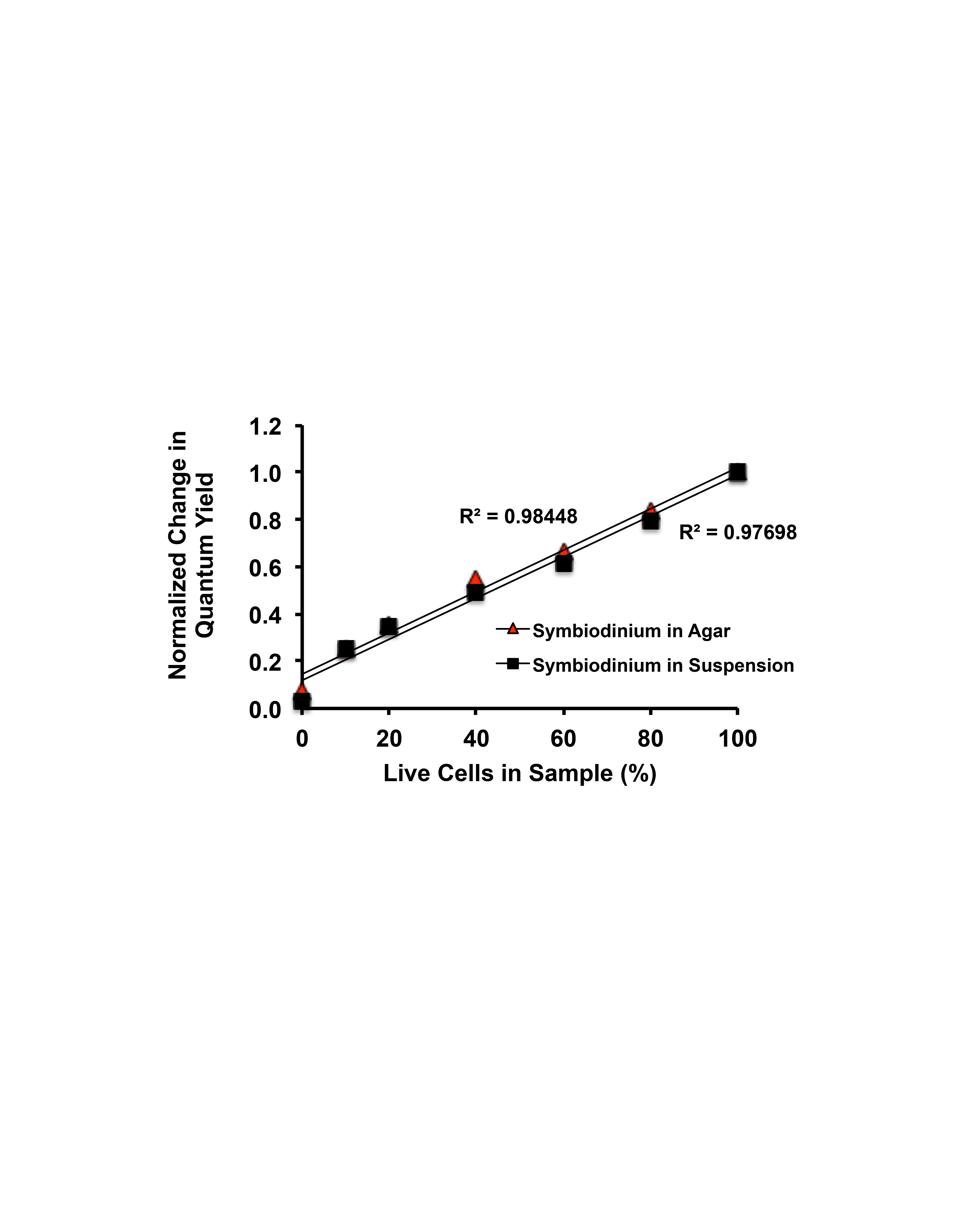

Supplement: S1 Fig — (TIF) [file pone.0136358.s001.tif]

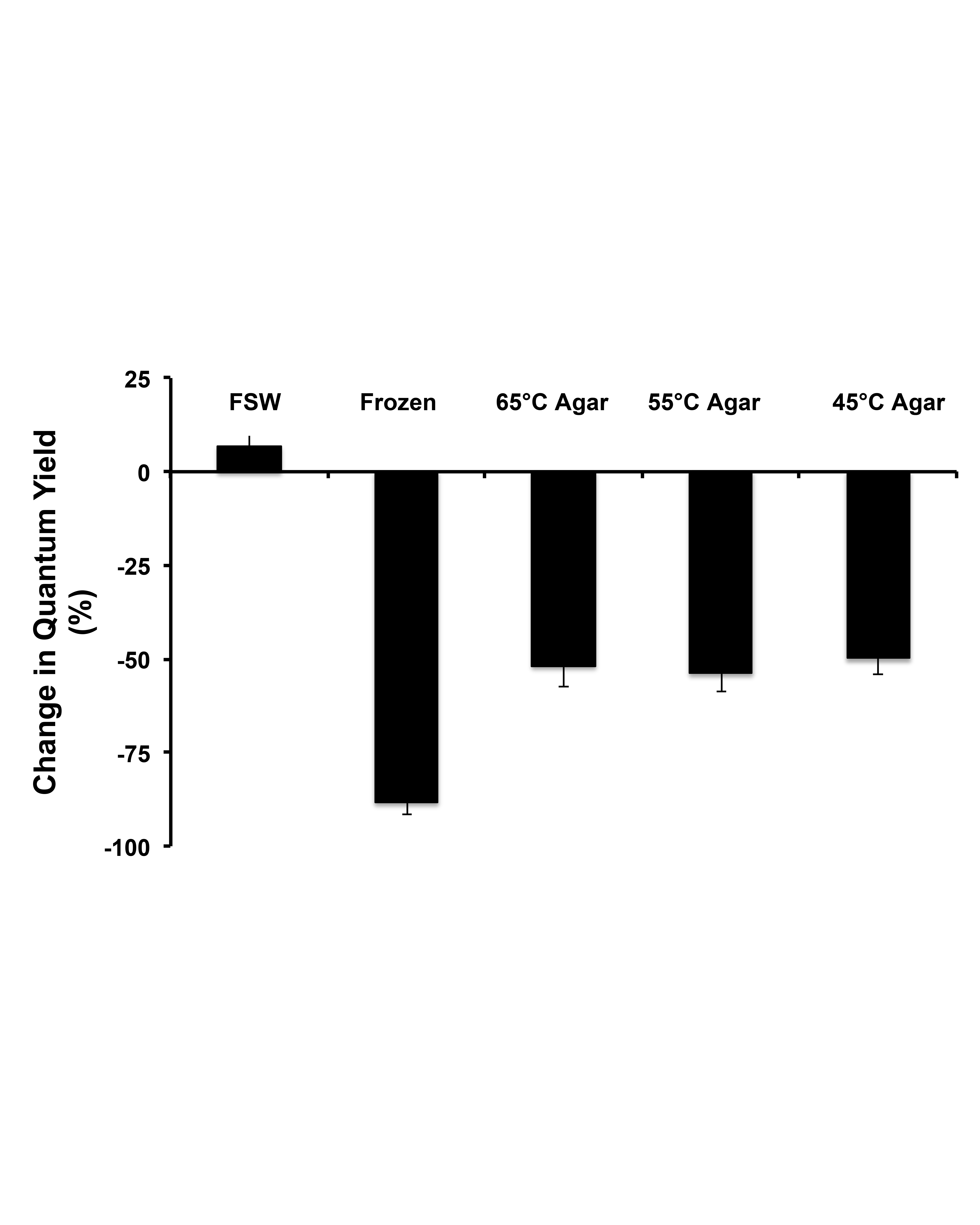

Supplement: S2 Fig — None of the temperatures tested affected quantum yield of the Symbiodinium after vitrification and 24 h maintenance in FSW (P > 0.05). (TIF) [file pone.0136358.s002.tif]

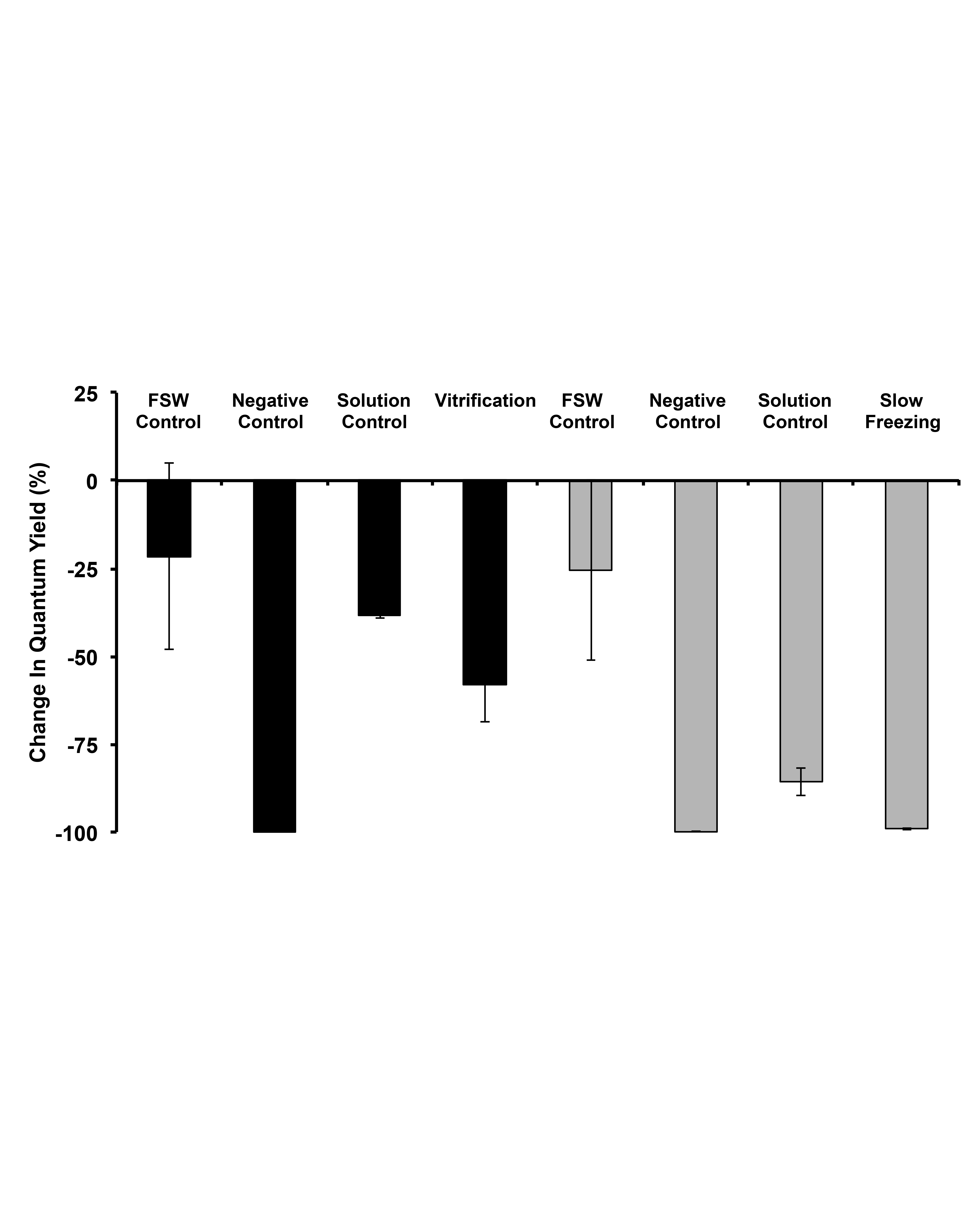

Supplement: S3 Fig — Only vitrification produces live cells post-thaw. (TIF) [file pone.0136358.s003.tif]

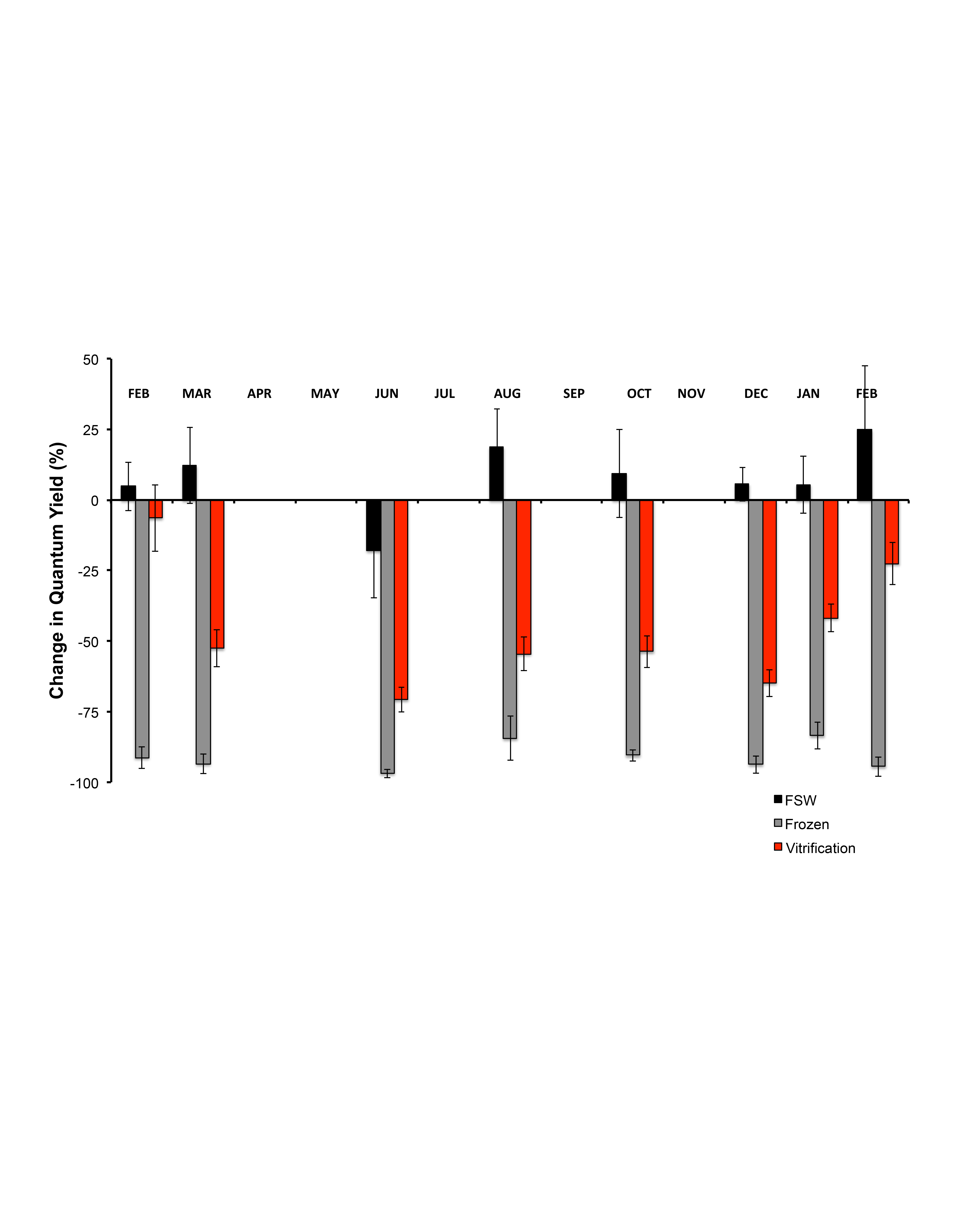

Supplement: S4 Fig — Winter months in Hawaii produced the best vitrification success. (TIF) [file pone.0136358.s004.tif]
